# The costs outweigh the benefits: seeing side-effects online may decrease adherence to statins

**DOI:** 10.1186/s12911-020-01207-w

**Published:** 2020-08-20

**Authors:** Nickolas M. Jones, Dana B. Mukamel, Shaista Malik, Robert S. Greenfield, Andrew Reikes, Nathan D. Wong, Emilie Chow

**Affiliations:** 1 Department of Psychological Science, University of California, Irvine, Irvine, CA USA; 2 Department of Medicine, Division of General Internal Medicine, iTEQC Research Program, University of California, Irvine, Irvine, CA USA; 3 Department of Medicine, Division of Preventive Cardiology and Cardiac Rehab, University of California, Irvine, Irvine, CA USA; 4 Department of Medicine, Division of Cardiology, University of California, Irvine, Irvine, CA USA; 5 Department of Medicine, Division of General Internal Medicine, Endocrinology, Diabetes, and Metabolism, University of California, Irvine, Irvine, CA USA; 6 Heart Disease Prevention Program, Division of Cardiology, University of California, Irvine, Irvine, CA USA; 7 Department of Medicine, Division of Internal Medicine/Pediatrics, University of California, Irvine, Irvine, CA USA

**Keywords:** Health-information seeking, Health decision-making, Statins, Medical websites

## Abstract

**Background:**

The prevalence of medical misinformation on the Internet has received much attention among researchers concerned that exposure to such information may inhibit patient adherence to prescriptions. Yet, little is known about information people see when they search for medical information and the extent to which exposure is directly related to their decisions to follow physician recommendations. These issues were examined using statin prescriptions as a case study.

**Methods:**

We developed and used a tool to rank the quality of statin-related web pages based on the presence of information about side effects, clinical benefits, management of side effects, and misinformation. We then conducted an experiment in which students were presented with a hypothetical scenario in which an older relative was prescribed a statin but was unsure whether to take the medication. Participants were asked to search the web for information about statins and make a recommendation to this relative. Their search activity was logged using a web-browser add-on. Websites each participant visited were scored for quality using our tool, quality scores were aggregated for each participant and were subsequently used to predict their recommendation.

**Results:**

Exposure to statin-related benefits and management of side effects during the search was significantly associated with a higher probability of recommending that an older relative adhere to their physician’s recommendation. Exposure to misinformation and side effects were not associated, nor were any other participant characteristics. Bigram analyses of the top reasons participants gave for their recommendation mirrored the statistical findings, except that among participants who did not recommend following the prescription order, myriad side effects were mentioned.

**Conclusions:**

Our findings suggest that units of information people see on health-related websites are not treated equally. Our methods offer new understanding at a granular level about the impact of Internet searches on health decisions regarding evidence-based recommended medications. Our findings may be useful to physicians considering ways to address non-adherence. Preventive care should include actively engaging patients in discussions about health information they may find on the web. The effectiveness of this strategy should be examined in future studies.

## Background

The availability of health information on the Internet has transformed the landscape of health provision in the United States. Increasingly, people with adverse health symptoms rely on Internet search engines to locate information about their symptoms [1, 2]. In 2013, 35% of adults in the U.S. indicated using the internet to learn more about medical conditions for themselves or someone else [1]. Some patients supplement doctor consultations by searching the internet for additional information [2], while others rely on the internet for self-diagnoses to determine whether they should initiate contact with their doctor [3]. Despite burgeoning attention to health-information seeking on the Internet, little is known about the downstream consequences of this behavior.

The literature on medical information seeking to-date has focused on two main questions: who engages in medical information seeking and how they go about doing so. Studies found that people with health anxiety [4], women [1, 5], young people, and highly educated and wealthy individuals are more likely to seek out health information on the Internet [1]. Researchers focused both on how people search for health-related information and how they synthesize that information to understand a particular diagnosis. For example, adolescent participants used trial-and-error to construct searches and sift through search results in an unsystematic way [6]. Others found that lower income individuals tend to search for more specific health-related information compared to those with higher income who conduct more general searches [7]. They also found that lower-income individuals rely on an intuitive (i.e., fast, unconscious) approach to processing health-related information learned from Internet searches, whereas higher-income individuals rely on a more deliberative (i.e., slow, conscious) approach [7].

An important area garnering much less attention is an examination of the outcomes associated with health information seeking. Work in this area tends to focus on perceived usefulness of the information patients find, their level of trust in the information sources they consult, and whether they will either initiate a physician visit or consult their physician again after conducting a search [8]. However, some evidence suggests that health information seeking is associated with lower adherence to medical recommendations [9, 10] as well as decisions about medical treatments [11]. In fact, there is a growing concern among providers that the increasing reliance on the Internet for medical information, coupled with what many perceive to be a high probability of encountering misinformation, would lead patients to make the “wrong” decisions about their own treatment and care [12, 13].

Although cross-sectional studies have examined the relationship between health information seeking and health decisions, none have examined whether the information people are exposed to during a health information search directly impacts decisions they make about treating their own health conditions. When faced with a concern over a standard medication, an internet search for related information can lead to exposure to information that varies in quality or veracity. Ideally, information seekers would find high-quality information based on scientific data from reputable health organizations. Unfortunately, the risk of being exposed to inaccurate and often unverified, or anecdotal information via social media, is nontrivial [14].

The classic example of misinformation that has gone viral on the internet and has caused undisputable and immeasurable public health harm is the case of vaccination. One unfortunate incident with misinformation linking autism to childhood vaccination [15] fueled a global social and political movement of “antivaxxers” that various governmental health organizations, such as the Center for Disease Control (CDC) and physician organizations have been fighting for many years now [16]. However, vaccines are far from being the only medical treatment that has been subject to misinformation, leading many to reject them despite the strong evidence-base supporting them and the recommendations of medical professionals, resulting in detrimental effects to the health of the public. Examples can be found in almost every area, ranging from cancer [17], to orthopedic [18, 19] to diabetes [14]. A better understanding of internet-based health-information seeking and its consequences is required.

In this paper, we chose to study statins because they are a widely prescribed medication, with a strong evidence-base supporting their use to prevent cardiovascular disease [20], but they are not well adhered to; one estimate indicates that as much as 58% of the U.S. population is non-adherent [21]. Moreover, there is controversy (and misinformation) about their associated risks and effectiveness. We first developed a tool to assess the quality of statin-related information on the Internet with input from an expert panel of clinicians. Then, we staged an experiment designed to a) track individuals as they searched for statin-related information on the Internet, b) quantify the quality of statin-related information participants saw using the tool we developed and c) determine whether information exposure was associated with a decision to recommend adherence to a statin prescription.

We note that in this experiment we chose to examine adherence to the recommendation of the physician for the initiation of statin treatment, rather than to decisions that patients already taking the medication might be making (i.e., lowering the dose or discontinuing treatment). The latter are often more complex decisions, reached due to side effects or cost considerations, and embody the actual experience of the patient with the medication, which we could not simulate in this study.

We hypothesized that exposure to information about side effects and to negative misinformation during the search would be associated with a lower likelihood of recommending the drug. On the other hand, exposure to benefits of statins and to the management of side effects would be associated with an increased likelihood of recommending the drug. We also used a natural language processing technique to analyze participants’ reasons for their recommendation.

## Methods

### Development of a tool to quantify the quality of the of internet information (CLARIFI)

We used a multi-step process, described below, to develop a tool (CLinically Applied Ratings to Internet-Found Information: CLARIFI Statins) measuring the quality of statin-related information on websites across the Internet.

#### Expert panel recruitment

We convened an expert panel comprised of cardiologists, primary care physicians, endocrinologists, lipidologists, and epidemiologists practicing in a medical school situated in a major U.S. university whose practice serves a diverse patient population ranging from academic personnel with employer insurance and high income, to non-English speaking immigrants covered by Medicaid and of low income families. All panel members had at least a decade of clinical experience.

#### Development of tool items

We performed an extensive literature and web search to identify factors found to influence patients’ decisions to adopt their physicians’ recommendation to begin a statin regimen or not. We created a list of both “true”, evidence-based facts (e.g., statins lower mortality) and misinformation (e.g., statins cause cancer). We consulted our expert panel to ensure that the list was comprehensive. We then reviewed the list with undergraduate students to ensure that the language describing each item was understandable to non-medical professionals and could be easily applied.

This process resulted in 40 items capturing whether a website provides information about side effects, benefits of statin medications, side effect management, and misinformation.

#### Development of numerical weights

We used the Delphi Method [22] to obtain panel consensus around relative weights (ratings) for each of the items. The Delphi Method includes several rounds in which each panel member responds to a survey about the relative weights. Following each round, the average weights for each item (where the average is calculated over all responses by panel member for the same item) are provided to the group with anonymous comments from members explaining the rational for their rating. Typically, consensus is reached after two or three rounds. We chose this approach because it allows each expert to express his or her opinion independently, without pressure of interpersonal relationships and personality conflicts.

At the start of this process, the panel was instructed to consider the importance of each CLARIFI item for a medically-focused website targeting patients seeking information about statins. We emphasized to the panel that the target audience for the website is the patient and not a professional audience. The panel rated the importance of each item on a scale from 0 (not important at all) to 10 (extremely important). To aid in the rating process, panel members were provided with a visual analog scale.

The results of the first round were summarized for each of the 40 items and presented to the panel. This summary included each item’s average weight calculated across all panel members, their ranges, and their coefficient of variations (defined as the variance divided by the mean for that item). Items for which consensus was reached during this round (with consensus defined as all panel member weights within +/− 20% of the mean) were removed from further consideration. For the second round, panel members were provided the summary information from the first round for the remaining items. They were also provided the +/− 20% range around the mean for each item, to facilitate their consideration. They were then asked to reconsider their initial weights, taking into account the summary information derived from the first round of responses. Each expert had the option to change their prior weight. Panel members were given an opportunity to explain their rationale for any weight they gave and encouraged to do so in particular if they chose an outlier weight compared with the average reported from round one. Group consensus on weights for each item was reached after three rounds of the Delphi process, except for 4 items. On these 4 items there was consensus among all members except one. Review of the comments and the variation in weights led us to conclude that further consensus on these four items was not likely. Table S1 identifies those 4 items in the footnote. The final CLARIFI – statins tool (see supplement Table S1) is based on the average consensus weights of the expert panel.

### Experiment recruitment and data collection

In the fall of 2017 and the spring of 2018, 212 undergraduate students were recruited and paid $20 dollars to participate in an experiment in which they were presented with a hypothetical scenario: An older relative who was prescribed by his or her physician with a cholesterol lowering medication (e.g., atorvastatin) is asking for advice. This relative is uncertain why the medication is needed and has heard from friends that it may be harmful. The relative knows that the student is in college, knows a lot about the Internet, and asks him or her for help in making a decision about whether to follow the doctor’s advice.

Each participant was given 90 min to search the Internet for information about statin medications and as they searched, a web browser add-on logged each website they visited and the duration of their visit.

### List of websites visited

Our aim was to generate a list of statin-related websites viewed by participants during the search period. The browser logger recorded that participants visited 16,931 Uniform Resource Locator (URLs or websites). After removing duplicate URLs across all participants, 6411 unique URLs remained. From these, we eliminated search engines (e.g., Google.com, Yahoo.com) and advertising redirect links using an automated R script. This resulted in a list of 2850 unique URLs that participants actually saw during the search protocol (and were potentially statin-related).

### Coding visited websites using CLARIFI

We determined inter-rater reliability by first training two lead research assistants to use CLARIFI. They calibrated their ratings on 40 randomly selected statin-related websites. Next, an additional ten coders and three medical residents were trained and calibrated on an additional 20 randomly selected websites.

Once coders were trained and calibrated, they were tasked with using CLARIFI to dichotomously code for the presence of each CLARIFI item (1 = information was present; 0 = information was absent) among all 2850 unique URLs. Many of these unique URLs contained irrelevant information, expired links, or led to academic articles rather than general consumer information and were not codable with CLARIFI.

The final number of codable statin-related websites directed at consumers was 980 (34.3%). We calculated kappa statistics (*κ* [23];) to determine the degree of chance-corrected agreement between the coders on each of the 40 CLARIFI items. We then multiplied each dichotomously coded item with the CLARIFI weighted scores derived from the Delphi process.

### Post-search survey variables

After completing their Internet search, participants completed a survey to a) indicate whether they decided to recommend that their relative take the medicine and b) describe their reasoning for their recommendation.

#### Dependent variable

At the end of the search task participants were asked “Given what you know about this drug, what recommendation would you make to your older relative?” Possible response options were “Tell your relative to use the drug” (coded 1), “Tell your relative not to use the drug” (coded 0).

#### Independent variables

##### Statin-related information exposure

We were interested in understanding the impact of the presence of different types of CLARIFI information on the decision to recommend. To assess this, we submitted the matrix of all 980 websites, with dichotomously coded CLARIFI items, to a factor analysis using Stata 14.2 (Stata Corp, Texas) and employed the principal components factor option. We identified four factors corresponding to four information types: side effects, clinical benefits, side effects management, and misinformation. Factor loadings above 0.4 were retained in the rotated solution and CLARIFI items mapped on well to each content area (see supplement Table S1).

The factor loadings corresponding to each of the four factors were then used to predict content scores for each statin-related website. These content scores were merged in with the participant web-search logs. We then created four information exposure scores for each participant by averaging content scores across the four content areas by participant. These scores were available for 190 (89%) participants who visited websites that were codable using CLARIFI.

The 22 participants with missing scores were those who visited websites that we could not access due to special university access requirements. These participants did not differ from the complete sample in any meaningful way.

##### Relative gender

Because side effects of statins may be different for men and women, the survey asked participants to indicate the gender of the elderly relative they imagined for this task. Most participants imagined a female relative (*n* = 135; 74%).

##### Sample demographics

The survey asked participants to provide information about their gender, ethnicity, as well as income level rated on a ladder graphic that ranged from 1 (income is well below the average American) to 10 (income well above the average American), whether they were born in the United States (1 = Yes, 0 = No), and whether English is their primary language (1 = Yes, 0 = No). Two participants had missing information on some demographic items and were subsequently removed from the sample.

#### Top three reasons for recommendation

After participants made their recommendation, they provided their top three reasons for their recommendation in three separate open-ended text fields. Responses across these three fields were concatenated and a bigram analysis was conducted in R Software [24] using the *tidytext* package [25] to extract prevalent themes. First, words in the corpus were tokenized into adjacent word-pairs (i.e., bigrams). For example, the sentence “I wish I could fly” would be broken into the following word-pairs: I wish, wish I, I could, could fly. Next, stop words, or commonly used words that do not convey information about content (e.g., the, and), were removed from the corpus of participant generated text. Bigrams were then generated by first counting all matching words-pairs among participants who recommended the drug and then by those who did not. Group-specific bigrams were then assigned an importance weight using the term frequency-inverse document frequency (tf-idf) function in *tidytext*, a common technique that weighs less commonly used words in the English language more heavily than more commonly used words. The top 20 bigrams (i.e. word-pairs) for each group were retained. This process was repeated after the words in this corpus of text were stemmed using the *SnowballC* package [26] in R to reduce each word to its root unit (i.e., prevented and preventing become prevent).

#### Analytic strategy

All statistical analyses were conducted in Stata 14.2 (College Station, TX) whereas visualizations and natural language processing were conducted using R. First, we assessed the reliability of the CLARIFI tool across human raters by computing a *κ* statistic. We then descriptively examined the distribution of CLARIFI scores across all 980 coded websites. We also averaged the CLARIFI ratings by *root* websites. For example, individual web pages hosted by WebMD.com in our data set each had a CLARIFI score; we averaged CLARIFI scores across all the web pages associated with WebMD.com to create a CLARIFI score for WebMD.com, the root website.

Next, a logistic regression model was estimated using the independent variables listed above to examine predictors of recommending the prescribed statin. We examined the data for outliers using Cook’s D and excluded 6 observations with Cook’s D values exceeding the critical value (based on our sample size) of 0.022 [27]. The results we present are based on 182 observations.

In our last analysis, we used a natural language processing technique (i.e., bigram analysis) to qualitatively examine the content of participants’ reasoning for the recommendation they gave. We analyzed the top bigrams by decision-group and assessed for each bigram’s level of importance. Top bigrams were then compared with the results of the logistic regression to ascertain whether participant reasoning corresponded to the information they were exposed to during their Internet search.

## Results

### Sample characteristics

The sample was comprised primarily of women (*n* = 132, 72%) and the majority of participants thought of a female relative (74%). The income level of our sample was 5.5 (*SD* = 1.8) on a 1–10 scale, indicating that, on average, respondents were middle income. The majority of participants were U.S. born (74%), of Asian descent (39%), followed by Latinx (23%), and Whites (16%). Less than half of participants (44%) did not speak English at home.

### Inter-rater reliability of CLARIFI items

The *κ* statistics by item are presented in Supplement Table S2. This statistic ranges from 0 to 1; values closer to 0 mean very low agreement between raters and values closer to 1 mean high agreement [23]. The *κ* statistics we calculated ranged from .42 to 1. Six items do not have a *κ* calculated because there was no variation across the websites, i.e. all websites either did or did not have the characteristic referred to in the item. The other thirty-four items had the following distributions: 12 items, including all the misinformation items, showed perfect agreement with *κ* = 1, 6 items had very strong agreement with κ > .80, 11 items exhibited substantial agreement with .60 < *κ* < .79. Only 5 items had moderate agreement with .40 < κ < .59. This level of high overall inter-rater reliability suggests that CLARIFI is a reliable rating tool.

#### Information quality across statin-related websites

**Fig. 1:**
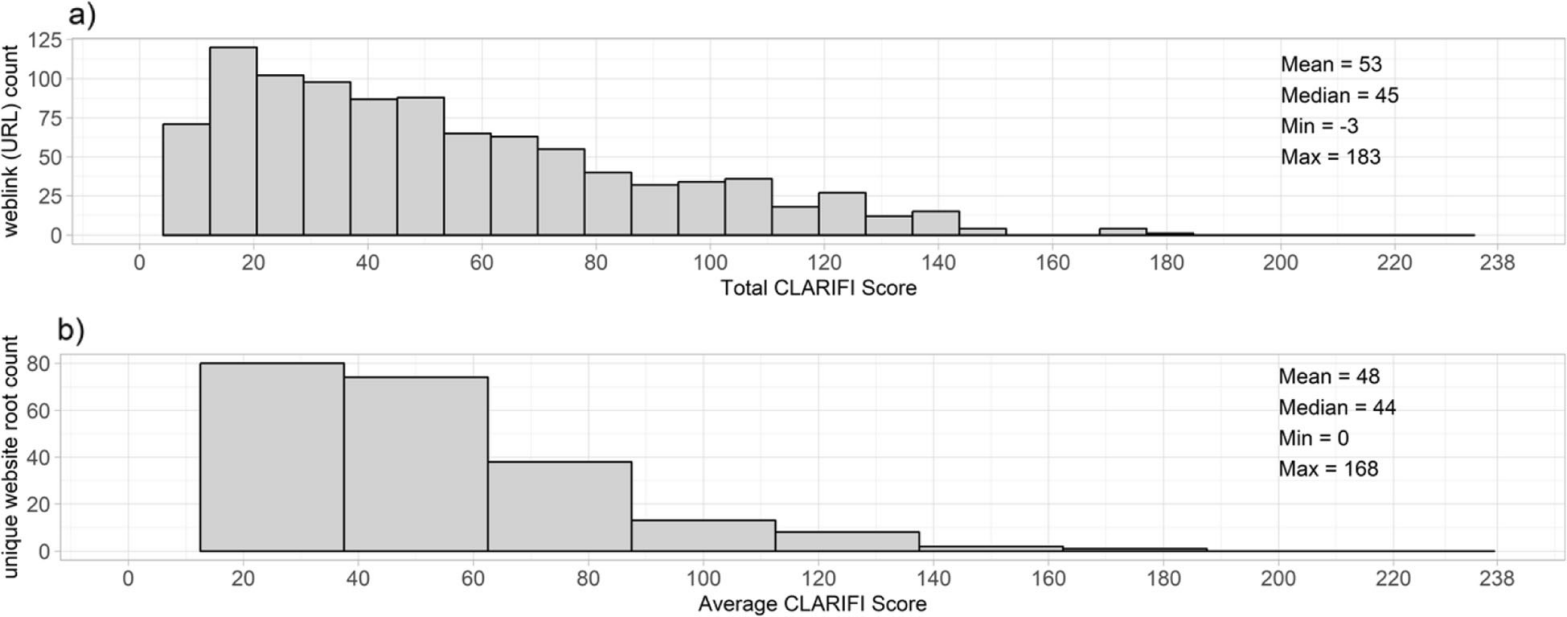
Distributions and summary statistics of CLARIFI scores across **a** 980 unique weblinks (URLs) and **b** averaged across 231 unique root websites

Figure 1 presents a) the distribution of CLARIFI total scores for the 980 URLs that were coded and b) the CLARIFI scores that were averaged across URLs for the 231 root websites associated with these URLs. The average URL score was 53, the median was 45, the minimum was 3 and maximum was 183. Compared to the maximum attainable score for a URL that meets all the CLARIFI criteria (238.6), an average of 53 suggests that the majority of URLs are lacking a substantial amount of information deemed important by the expert panel. Moreover, a median of 45 implies that 50% of URLs have less than 19% (45/238.6) of the optimal informational content suggested by CLARIFI. Clearly, most websites provide minimal information to the general public. The best URL with the most information reached only 77% of the maximum CLARIFI information score, still about 25% below the maximum.

**Fig. 2:**
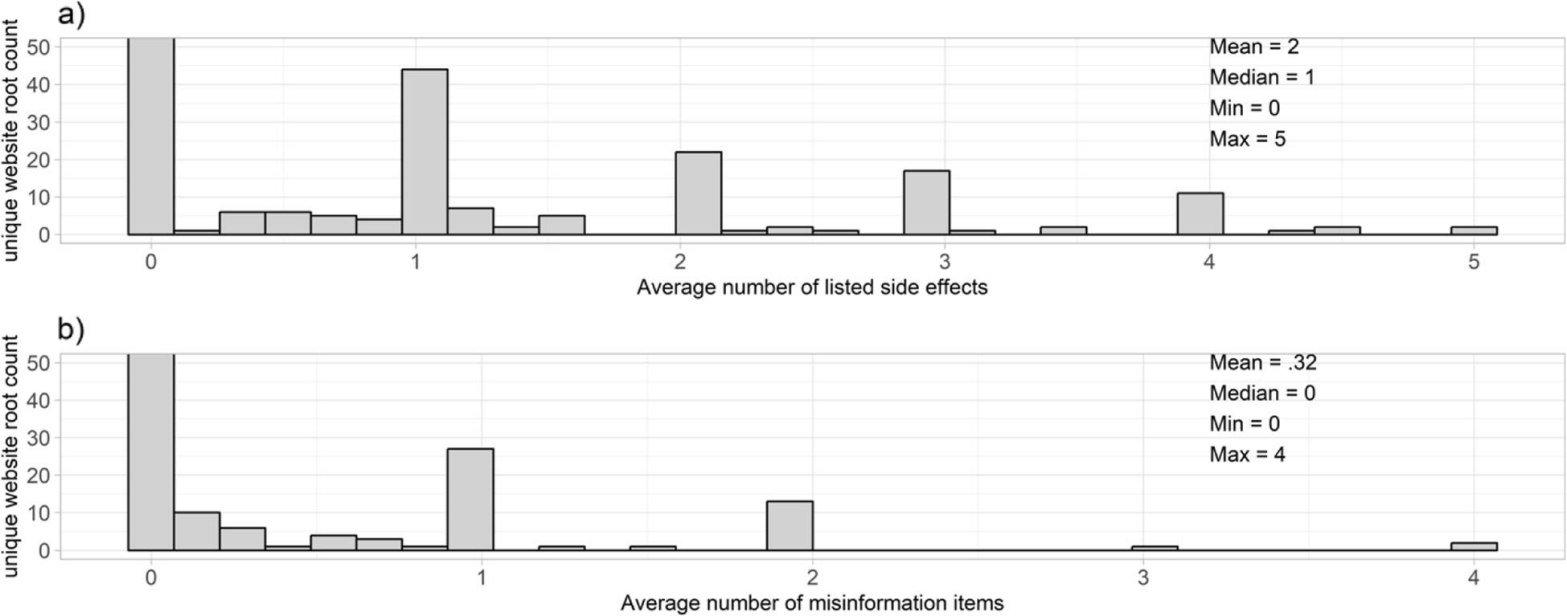
Distributions and summary statistics of **a** true side effects and **b** misinformation across 231 unique website roots. *Note:* The y-axis stops at 50 to allow for an accurate visual comparison between both graphs

Figure 2 presents the distribution of true side effects (CLARIFI items 10–14) and misinformation (CLARIFI items 15–18) that are discussed in the 231 root websites. The average number of side effects items per root website was 2 (Median = 1, Min = 0, Max = 5). The average number of misinformation items per root website was 0.32 (Median = 0, Min = 0, Max = 4). Sixty-one percent (*n* = 142) of root websites mentioned side effects and 22.5% (*n* = 52) contained misinformation about statins.

#### Survey descriptive statistics

On average, participants spent 57 min (*SD* = 20) on the search and survey and, on average, visited 72 websites (*SD* = 58). Neither the duration of their search nor the number of websites visited were associated with the decision outcome. Descriptive statistics for all independent variables are presented in Table 1. Forty-one percent of participants (*n* = 75) recommended that their older relative take a statin. Across the sample, average statin-related factor scores ranged from − 1.81 to 4.41.

**Table 1:** Descriptive statistics of all model variables (*n* = 182)

Variables	Mean	SD	Min - Max
Decision to recommend taking statin (yes = 1)	.41		0–1
*Exposure to information about*			
Side effects	.21	.68	−1.81 – 2.99
Benefits	.26	.52	−.88–2.23
Management	.18	.67	−.92–4.41
Misinformation	.28	.55	−.83–2.48
Income level	5.56	1.86	1–10
	**Count (%)**		
Gender of relative			
Female	135 (74%)		
*Sample Demographics*			
Female	132 (73%)		
Ethnicity			
Asian	71 (39%)		
Latinx	42 (23%)		
European American/White	30 (16%)		
Other	39 (22%)		
Born in U.S.	134 (74%)		
English language spoken at home	102 (56%)		

#### Logistic regression

Table 2 presents the logistic model predicting the decision to recommend taking a statin. Exposure to information about side effects (*p* = 0.43) and to misinformation (*p* = 0.29) about statins were not related to participants’ decision to recommend the drug. However, exposure to information related to the benefits of statins and information related to management of side effects both significantly increased the likelihood of a decision to recommend to a hypothetical relative that they take statins when controlling for all variables in the model.

**Table 2:** Logistic regression predicting the decision to recommend that an older relative take a statin (*n* = 182)

*Variables*	*OR* [CI]	*p value*
Side effects	1.22 [.74–2.01]	.43
Benefits	2.07 [1.07–3.98]	.02
Side-effects management	1.90 [1.06–3.41]	.02
Misinformation	1.38 [.74–2.55]	.29
Relative gender (female = 0)	1.55 [.73–3.28]	.24
Participant gender (female = 0)	1.26 [.60–2.64]	.53
Race/Ethnicity (white = 0)		
Latinx	.62 [.19–1.94]	.41
Asian American	.80 [.30–2.11]	.65
Other	1.09 [.34–3.4]	.87
Born in the U.S. (no = 0)	.50 [.20–1.23]	.13
English spoken at home (no = 0)	.55 [.24–1.26]	.15
Income level	1.07 [.88–1.31]	.45

Model statistics: Model pseudo *R*^2^ = .10; *χ*^2^ = 25.77, *p* = .01

Note: *p < .05; *OR* Odds ratio, *CI* Confidence interval

Interestingly, the other covariates typically found to be associated with health decisions, including income level, educational status, primary language, gender or gender of the relative, were not associated with participant recommendation.

#### Bigram analysis - top reasons for recommendation

**Fig. 3:**
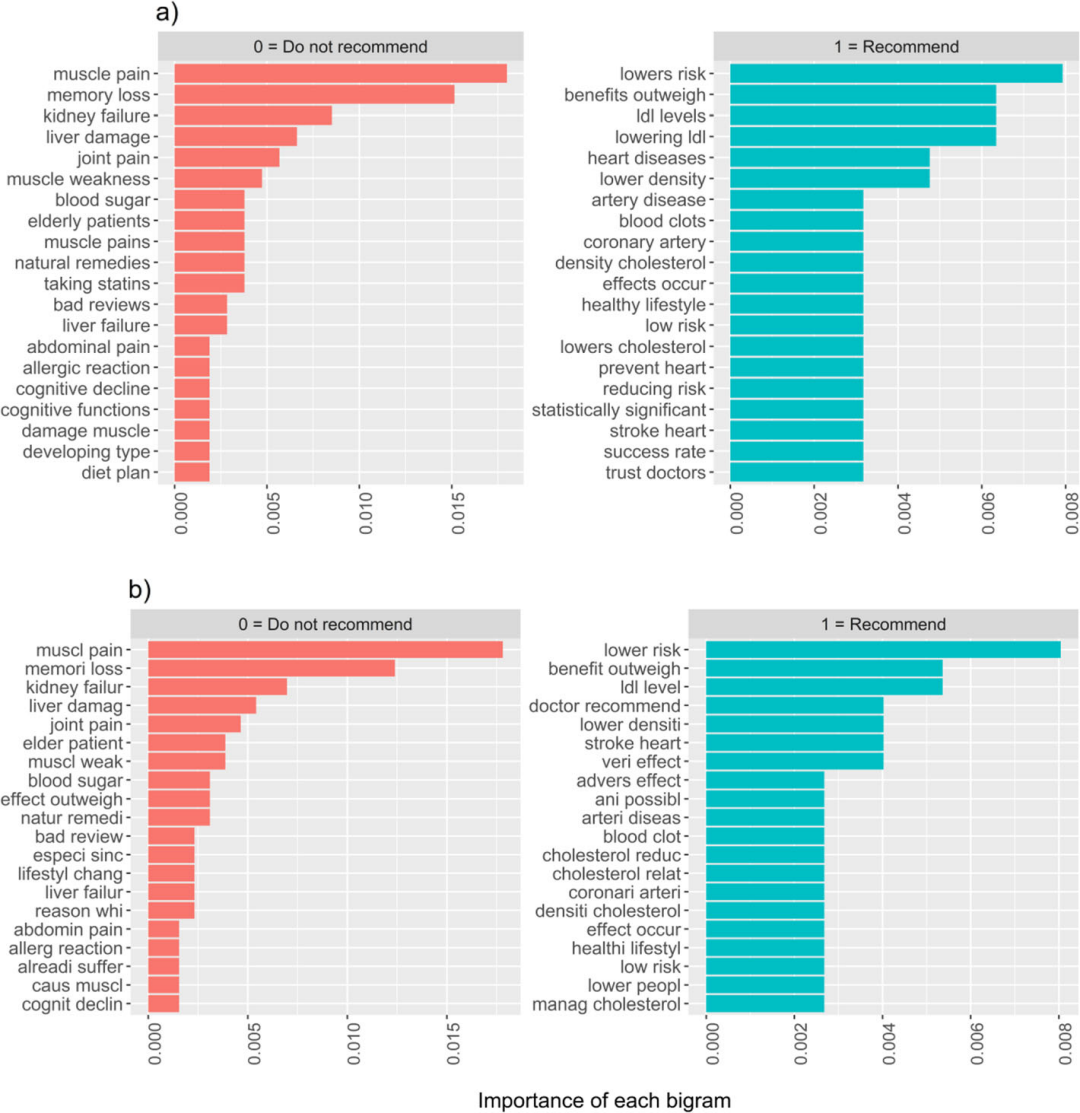
Bigram analyses of **a** raw and **b** stemmed text of the top reasons participants gave for their decision whether (or not) to recommend that their older relative take a statin as prescribed

Consistent with the logistic regression findings, the bigrams revealed that participants who recommended that their older relative take a statin focused on the benefits of statins (e.g., lowering cholesterol, prevent heart attacks and cardiovascular disease; see Fig. 3) and specifically stated that the “benefits outweigh the risks.” Although exposure to information about side effects was not significantly associated with the decision participants made, those who did not recommend the drug focused primarily on the potential side effects (e.g., muscle pain/weakness, memory loss, and kidney failure).

## Discussion

Using a mixed-methods approach involving an information quality assessment tool and quantitative and qualitative analyses, we studied how exposure to statin-related information on the Internet is associated with decision-making in a hypothetical context. First, after developing a tool to quantify the quality of statin-related information on the Internet, we found that 22.5% of root websites visited by participants contained misinformation and 50% of sites provided only minimal information about statins, offering less than 19% of the content expected by CLARIFI, and none exceeding 77% of the optimal informational content. Despite the relatively low prevalence of misinformation across websites (22.5%), nearly 60% of participants recommended that their relative ignore their statin prescription.

Overall, our analyses indicated that exposure to information about the clinical benefits of statins and management of potential side effects were significantly associated with a decision to recommend that an older relative initiate a statin treatment. An analysis of the reasons each participant gave for their decision reflected, for the most part, the “weight” of the information they saw online: Recommenders focused on the benefits while non-recommenders focused on the side effects of statins.

These findings suggest that units of information people see on health-related websites are not treated equally by consumers. Generally, the health-related information people are likely to encounter has been found to be of high quality [28], although more recent evidence suggests otherwise [12]. However, the factors that lead one person to focus on negative potentials versus clinically supported positive outcomes are not well understood. Some researchers posit that health anxiety may lead people to feel more worried about medical symptoms after an internet search [29]. Yet, other processes may also be implicated. For example, the search engine and terms people use to begin their search can be markedly different (although 98% of individuals in our sample used Google). This means that as a function of the person conducting the search and terms they input, the trajectory of a search can take myriad tracks that may lead to exposure to more negative information than positive or vice versa. Examining the search process itself deserves more attention and may potentially be a strong mediator of health decision making.

Our findings, taken together with other studies suggesting that patients are influenced by information they read on the Internet and are often influenced by it more than they are by the information provided by the medical providers, indicate that the field has to consider a more expansive notion of the concept of preventive care. No longer is it sufficient for providers to talk to their patients about what they ought to do to prevent disease and maintain health, or to prescribe medications and treatments that enhance health. With the advent and “democratization” of medical-related information, be it correct or incorrect, providers need to identify ways to “immunize” their patients against the influence low quality information on the Internet. Our study does not suggest how this can be accomplished, but it does begin to show that not all areas of information are equally influential.

It is likely that interventions to “immunize” patients against the Internet will take the form of some communication and educational component of the patient/provider encounter, dedicated to a discussion of this issues as it relates to the specific medical condition of the patient at the time of the encounter. However, in the reality of a busy clinical practice these days, and the current reimbursement environment, it is unlikely that the vast majority of providers will be able to allocate the time to such an intervention which is likely to be time intensive. It is, therefore, important to understand what patient types are most likely in need of such interventions and what strategies are most likely to be successful in convincing them to seek credible, evidence-based information.

We note several limitations to our study. First, our results revealed that exposure to misinformation about statins had no bearing on decision making, however, we did not fully capture exposure to other potential sources of misinformation on social media. For example, the CLARIFI tool was not designed to evaluate YouTube videos and message boards (e.g., Reddit) that participants in our sample visited and hence these were excluded from our analysis. Thus, we might have underestimated the negative information our subjects were exposed to.

Second, our study sample (university students) is not reflective of patients typically prescribed statins (i.e., older adults). Individuals in our sample are more likely to be Internet literate than the general population and they are also trained in searching for scientific evidence, with unfettered access to the university’s library database. Although conducting a similar study with a patient population would be more ecologically valid, our approach mitigated certain ethical considerations implicated in studying patients who are prescribed a statin for the first time. The introduction of an Internet search to an actual patient who may not have performed a search otherwise is an intervention which may change that patient’s decision to adhere to their prescriber’s recommendation. Thus, an experiment and intervention such as this may harm patients, and hence is not ethical.

Third, our experiment presented subjects only with the decision to recommend that an older relative start a statin treatment. We did not investigate decisions faced by patients already in treatment who may be considering lowering the recommended dose or stopping treatment all together. It is likely that our findings about the impact of exposure to statin-related information on the Internet will generalize to these other decision points. However, it is possible that information exposure, coupled with personal experiences with statins, may amplify or reduce the weight patients ascribe to different information types (i.e. benefits, side effects, management, misinformation).

An additional limitation is that in our analysis, we treated each information type independently and did not account for the fact that many participants saw most information types on the same webpage. Unfortunately, our sample size did not allow us to examine potential interactions between different information dimensions. Future work, with a larger sample, should examine the interplay between the types of information people are exposed to when conducting a health information search.

We find it surprising that no demographic factors influenced participants’ recommendations. It is possible that undergraduate students from the same university are too homogenous, or perhaps that we did not measure those factors that would have influenced their decisions differentially. Future work should seek an older participant population, while balancing the ethical concerns noted earlier.

This last limitation raises concern about generalizing from this study. Yet, we note that the percent of student participants who chose to recommend use of a statin in our study (41%) is very similar to the national average of patients adhering to their provider’s recommendations to take statins (42% [21];). This might suggest that, and at least with respect to the impact of information, this study can be viewed to offer initial hypotheses to be tested further in the future.

There is a significant increased risk of dying with lack of statin adherence among patients with atherosclerotic heart disease [30]. Our findings highlight the potentially lethal perspective over 50% of our subjects come away with after engaging in an Internet search about statins. Moreover, they raise questions about who these 50% are and what about the information on the Internet convinces them to eschew the advice of traditional providers. Are these individuals driven by the true, yet unpleasant, facts about side effects that are not simultaneously accompanied by information about side-effects mitigation? Is it exposure to misinformation, which we surprisingly found to be less prevalent than we expected? Is it a more general mistrust of the medical profession [31]? Or are the decisions these individuals make driven by the missing information that might have swayed opinions in the direction of a better decision had it been present? More studies like the one we present, addressing some of the limitations our study faced, should pave the way to the design of novel interventions that would assist in answering these important questions and helping providers to lead their patients to medical websites that would enable them to make evidence-based choices.

## Conclusion

Our findings suggest that statin-related websites vary widely in the quality of consumer-facing information they present. They also suggest that individuals engaging in a search of statin-related information are not likely to treat pertinent information equally, differentially weighting the information that informs their medical decisions. The granularity of our methods offers new understanding about the impact of Internet searches on health decisions regarding evidence-based recommended medications. We believe that our results may be useful to physicians considering ways to address non-adherence. In particular, preventive care might acquire a new component, actively engaging patients in discussions about health information they may find on the web. The effectiveness of this strategy should be examined in future studies.

## Supplementary information

**Additional file 1:** **Supplemental Material.** The supplemental material includes details of the CLARIFI STATINS tool (Table S1) and information about factor loadings in four information-content areas across CLARIFI items (Table S2).

## Data Availability

Data and materials are available upon request from the corresponding author.

## Abbreviations

CLARIFI: Clinically Applied Ratings to Internet-Found Information
CDC: Centers for Disease Control
URL: Uniform Resource Locator
