# Supplementary material for: The costs outweigh the benefits: seeing side-effects online may decrease adherence to statins
Source: BMC Med Inform Decis Mak. 2020 Aug 20;20:197. doi: 10.1186/s12911-020-01207-w (PMC7439707; doi:10.1186/s12911-020-01207-w)
Supplement: Supplementary file 1 — Additional file 1: Supplemental Material. The supplemental material includes details of the CLARIFI STATINS tool (Table S1) and information about factor loadings in four information-content areas across CLARIFI items (Table S2). [file 12911_2020_1207_MOESM1_ESM.docx]

**Table S1.** CLARIFI tool instructions, items, and weights.

| ***CL****inically* ***A****pplied* ***R****atings to* ***I****nternet-****F****ound* ***I****nformation - STATINS* ***(CLARIFI STATINS)*** | | | | | |
| --- | --- | --- | --- | --- | --- |
| **Instructions:** The objective of CLARIFI is to assess the value of web sites about statins in educating patients about evidence-based information. CLARIFI was designed by an expert panel including cardiologists, endocrinologists, and internists. To use CLARIFI, review the website for the presence of each item and enter the "1" in *Column 5* for each criterion that is met by the website. CLARIFI will apply the weights and calculate an overall score for the site. The maximum score of a website meeting all criteria is 238.6. | | | | | |
| **Item #** | **CLARIFI Items (and sub-items)** | | **Delphi Weight** | **Enter 1 if response is YES; 0 or blank if NO** | **Item score** |
| 1 | Does the website state explicitly that statins lower “bad cholesterol”? LDL not necessary to state – but not discourage | | 8.4 |  | 0 |
| 2 | Does the website list clinical benefits of statins? | Lower mortality/death | 9.4 |  | 0 |
| 3 |  | Fewer heart attacks | 9.6 |  | 0 |
| 4 |  | Fewer strokes | 9.0 |  | 0 |
| 5 |  | Lower risk of heart disease | 9.2 |  | 0 |
| 6 |  | Lower risk of cardiovascular, vascular or blood vessel disease | 8.2 |  | 0 |
| 7 | Does the website advise on risk assessment for eligibility for statins? | Advises patients to review their personal risk reduction with their health care provider | 7.0 |  | 0 |
| 8 |  | Links to risk calculator | 3.0 |  | 0 |
| 9 | Does the website describe “How statins work”? |  | 5.0 |  | 0 |
| 10 | Does the website provide a balanced discussion of potential side effects of statins by stating that: | Statins may cause muscle pain | 7.6 |  | 0 |
| 11 |  | Statins may cause muscle weakness | 5.6 |  | 0 |
| 12 |  | Statins may raise blood sugar | 5.8 |  | 0 |
| 13 |  | Statins may cause muscle liver test abnormalities** | 4.4 |  | 0 |
| 14 |  | Statins may cause fatigue | 2.8 |  | 0 |
| 15 |  | Statins cause dementia or memory loss | -6.8 |  | 0 |
| 16 |  | Statins cause cancer | -7.8 |  | 0 |
| 17 |  | Statins cause confusion | -6.9 |  | 0 |
| 18 |  | Statins cause cataracts | -5.8 |  | 0 |
| 19 |  | Statins do not cause dementia or memory loss | 6.8 |  | 0 |
| 20 |  | Statins do not cause cancer | 7.8 |  | 0 |
| 21 |  | Statins do not cause confusion | 6.9 |  | 0 |
| 22 |  | Statins do not cause cataracts* | 5.8 |  | 0 |
| 23 | The website makes no specific, quantifiable statements about any side effect risks | | 5.2 |  | 0 |
| 24 | Does the website state that: | Statins are often a life-long therapy? | 6.4 |  | 0 |
| 25 |  | Statins should be stopped only after the patient discusses it with their health care provider? | 6.6 |  | 0 |
| 26 |  | Dose and frequency should be changed only after the patient discussed it with their health care provider? | 7.6 |  | 0 |
| 27 | Does the website clearly state that side effects can be managed? | By switching to another statin | 7.0 |  | 0 |
| 28 |  | By changing to another class of lipid lowering medications | 6.4 |  | 0 |
| 29 |  | By taking Co-Enzyme Q10 | 3.4 |  | 0 |
| 30 |  | Advising the patient to review their side effects with their prescribing health care professional | 7.7 |  | 0 |
| 31 |  | Advising patients that often side effects can be diminished or eliminated with treatment by their health care professional | 8.0 |  | 0 |
| 32 |  | Suggest to patients that symptoms may be due to other causes than statin and should be discussed with their prescribing health care professional | 7.8 |  | 0 |
| 33 | Side effect management advice is presented in proximity to the listing/discussion of side effects, and is given same visibility (e.g. font size) so that patients are likely to see other options when reading about side effects? | | 7.0 |  | 0 |
| 34 | Does the website encourage patients to make their decision about taking statins based on a balanced consideration of the benefits from taking them (e.g. less mortality) versus the risks (potential side effects)? | | 7.2 |  | 0 |
| 35 | Does the website suggest discussing cholesterol lowering treatment or therapy with the patient’s healthcare provider? | | 7.4 |  | 0 |
| 36 | Does the website discuss healthy lifestyle (including diet and exercise) as a complement or adjunct to statins (emphasize: not as an alternative to statins) | | 8.8 |  | 0 |
| 37 | Does the website state what is it’s the source of funding? *** | | 5.0 |  | 0 |
| 38 | Does the website provide references for the information provided? **** | | 4.6 |  | 0 |
| 39 | Does the website indicate when it was last updated? | | 4.8 |  | 0 |
| 40 | Does the website disclose conflicts of interest? | | 5.4 |  | 0 |
|  |  |  |  |  |  |
|  |  |  | **TOTAL CLARIFI SCORE** | | **0** |
|  |  |  |  |  |  |
| * | One expert panel member disagreed with the weight and assigned this item a weight of 2. | | | | |
| ** | One expert panel member disagreed with the weight and assigned this item a weight of 7. | | | | |
| *** | One expert panel member disagreed with the weight and assigned this item a weight of 8. | | | | |
| **** | One expert panel member disagreed with the weight and assigned this item a weight of 7. | | | | |

| **Table S2.** Factor loadings of CLARIFI items on 4 content areas of side effects, clinical benefits, misinformation, and side effects management | | | | |
| --- | --- | --- | --- | --- |
| CLARIFI Items | *Factor 1*  Side effects | *Factor 2*  Clinical benefits | *Factor 3*  Misinformation | *Factor 4*  Side effects management |
| Advises patients to review their personal risk reduction with their health care provider | .53 | - | - | - |
| Statins may cause muscle pain | .50 | - | - | - |
| Statins may cause muscle weakness | .48 | - | - | - |
| Statins may cause fatigue | .53 | - | - | - |
| Statins do not cause dementia or memory loss | .46 | - | - | - |
| Statins do not cause cataracts | .56 | - | - | - |
| The website makes no specific, quantifiable statements about any side effect risks | .56 | - | - | - |
| By switching to another statin | .65 | - | - | - |
| Advising the patient to review their side effects with their prescribing health care professional | .56 | - | - | - |
| Lower mortality/death | - | .57 | - | - |
| Fewer heart attacks | - | .45 | - | - |
| Fewer strokes | - | .75 | - | - |
| Lower risk of heart disease | - | .80 | - | - |
| Lower risk of cardiovascular, vascular or blood vessel disease | - | .61 | - | - |
| Lower mortality/death | - | .42 | - | - |
| Website describes how statins work | - | .55 | - | - |
| Statins cause dementia or memory loss | - | - | .69 | - |
| Statins cause cancer | - | - | .61 | - |
| Statins cause confusion | - | - | .69 | - |
| Statins cause cataracts | - | - | .69 | - |
| Statins cause dementia or memory loss | - | - | - | .42 |
| Statins are often a life-long therapy? | - | - | - | .61 |
| Statins should be stopped only after the patient discusses it with their health care provider? | - | - | - | .49 |
| By changing to another class of lipid lowering medications | - | - | - | .45 |
| By taking Co-Enzyme Q10 | - | - | - | .40 |
| Website suggests to patients that symptoms may be due to other causes than statin and should be discussed with their prescribing health care professional | - | - | - | .40 |
